# Endovascular Therapy Versus Best Medical Treatment in Posterior Cerebral Artery Stroke: A Systematic Review and Meta‐Analysis

**DOI:** 10.1002/brb3.71194

**Published:** 2026-01-07

**Authors:** Umama Alam, Rabia Ahmed, Shree Rath, Fazia Khattak, Muhammad Asad Asif, Monsurah Bisola Alatise, Lubaba Yunas, Adil Khan, Mohd Sijad Uddin, Labannya Das Puja, Sayed Inamullah, Mahin Fatima, Sumia Fatima, Fazeela Bibi, Abdul Moiz, Raheel Ahmed

**Affiliations:** ^1^ Khyber Medical College Peshawar Pakistan; ^2^ Jinnah Medical and Dental College Karachi Pakistan; ^3^ All India Institute of Medical Sciences Bhubaneswar Bhubaneswar India; ^4^ Healing House Psychiatric Clinic Rawalpindi Pakistan; ^5^ Faculty of Medicine and Pharmacy Cadi Ayyad University Marrakech Morocco; ^6^ Saidu Medical College Swat Pakistan; ^7^ NIMRA Institute of Medical Sciences, Jupudi Vijayawada Andhra Pradesh India; ^8^ Sir Salimullah Medical College Dhaka Bangladesh; ^9^ Rawalpindi Medical University Rawalpindi Pakistan; ^10^ Bacha Khan Medical College Mardan Pakistan; ^11^ Academic Clinical Lecturer Newcastle University Newcastle UK; ^12^ Department of Cardiology Freeman Hospital Newcastle upon Tyne UK; ^13^ Royal Brompton Hospital London UK

**Keywords:** endovascular thrombectomy, posterior cerebral artery, stroke

## Abstract

**Introduction:**

Posterior cerebral artery (PCA) stroke, while comprising a smaller proportion of ischemic strokes, often results in significant neurological deficits and poses distinctive therapeutic challenges. This study aims to systematically review and meta‐analyze the efficacy and safety of EVT compared with best medical management (BMM) in patients with PCA stroke.

**Methods:**

Electronic searches of PubMed, Embase, and Scopus up to July 2025 identified studies comparing EVT and BMM for PCA stroke. Eligible studies included patients with PCA stroke, compared EVT with BMM, and reported at least one outcome of interest. Statistical analyses employed random or fixed‐effects models as appropriate; results are presented as odds ratios (OR) or mean differences (MD) with 95% confidence intervals (CI).

**Results:**

A total of 12 studies were included, encompassing 39,525 patients (2,540 EVT; 37,075 BMM). EVT was associated with significantly increased odds of early neurological improvement (ENI) (OR 2.11, 95% CI 1.81–2.45), and greater reduction in NIHSS at 24 h (MD –1.30, 95% CI –1.89 to –0.71). There was no significant difference in visual field normalization and no difference in excellent functional outcome. EVT was associated with lower odds of achieving functional independence (mRS 0–2 at 90 days: OR 0.75, 95% CI 0.67–0.85), and significantly increased risks of symptomatic intracranial hemorrhage (OR 2.32, 95% CI 1.66–3.23) and mortality at 90 days (OR 1.86, 95% CI 1.47–2.35).

**Conclusion:**

In PCA stroke, endovascular thrombectomy (EVT) confers early neurological recovery but does not improve long‐term functional independence and is associated with higher risks of symptomatic hemorrhage and mortality compared with BMM. While EVT may benefit selected patients, these results underscore the need for individualized treatment decisions and prospective randomized trials focused on PCA stroke.

## Graphical abstract

Graphical abstract representing key findings of our study.

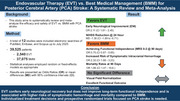

## 1 Introduction

The PCA is a paired terminal branch of the basilar artery, present in the circle of Willis. PCA provides supply to most parts of the occipital cortex, the inferior and medial temporal cortices, the thalamus, and rostral midbrain. PCA strokes comprise 6%–9% of all ischemic strokes (Nguyen et al. 2023). It often leads to debilitating visual, cognitive, and memory deficits, such as homonymous hemianopia, alexia without agraphia, or impaired recall, that substantially diminish quality of life (Nguyen et al. 2023). Yet, surprisingly, PCA infarction has received far less attention in research compared with anterior circulation strokes. Mostly, PCA occlusions are embolic in nature and arise from a cardiac source (24%), an arterial source (14%) or large artery atherosclerosis (32%) (Caplan et al. 2005). Other less frequent causes include arterial dissection, vacuities, fibro muscular dysplasia, and moyamoya disease (Kuybu et al. 2023). The pathophysiology behind PCA stroke is occlusion along the proximal PCA trunk, distal PCA trunk, or in its cortical branches. If there is proximal occlusion in the P1 or P2 segments, it would compromise blood to the thalamus and midbrain, whereas in distal occlusion, the calcarine or parieto‐occipital branches are affected, causing visual field impairment. The extent of ischemic injury is determined by collateral circulation, the posterior communicating artery, and individual vascular anatomy (Carrera et al. 2011).

The best medical treatment (BMT) for PCA stroke traditionally includes antiplatelet agents, anticoagulants, statins, and supportive care (Kuybu et al. 2023). Despite being considered the standard treatment, it has several evident drawbacks, such as difficulty in imaging salvageable tissue and persistence vessel occlusions (Carrera et al. 2011). Randomized controlled trials like MR CLEAN have demonstrated that EVT provides superior outcomes for anterior circulation large vessel occlusions, which lead to its incorporation into international guidelines as a Class I recommendation (Berkhemer et al. 2015; Powers et al. 2019). Hence, EVT is established as the standard of care for anterior circulation strokes. This successful advancement is leading to a growing interest in extending EVT to other territories, including the posterior circulation and more distal occlusions, such as the PCA. There have been reports of favorable outcomes in selected patients who had radiographically confirmed occlusions and had disabling symptoms. One of those includes the PLATO study, the largest study to date, which showed improved early neurological outcomes and visual recovery, albeit with higher risks of hemorrhage and mortality with EVT (Nguyen et al. 2023). Another supporting meta‐ analysis by Alkhiri et al. (2024) showed a significant early recovery (OR 2.31) and visual field normalization (OR 3.08) with EVT, though there were no differences seen in functional independence (Alkhiri et al. 2024).

However, these findings have yet to meaningfully influence clinical guidelines due to the lack of randomization and inconsistent criteria in performing EVT. While a previous meta‐analysis on EVT versus BMT in PCA stroke exists (Guo et al. 2025), it was limited by small sample sizes and heterogeneity across studies. Therefore, an updated systematic review and meta‐analysis (SRMA) is warranted to better inform clinical practice. Recognizing these gaps in the literature, we conducted an updated SRMA that incorporates newly published data, including a recent large‐sample study, to directly evaluate and compare key clinical outcomes in PCA stroke patients treated with EVT versus medical therapy. In addition, we assessed the certainty of the evidence using the GRADE framework and evaluated publication bias, thereby strengthening the robustness of our findings. By bringing together the latest and most comprehensive evidence with rigorous quality appraisal, our goal is to shed light on the real‐world effectiveness of these treatments, support clinical decision‐making, and highlight areas that need further research.

## 2 Methodology

### 2.1 Study Design and Protocol Registration

This SRMA was conducted in accordance with the guidelines set by the cochrane collaboration (Cochrane Handbook for Systematic Reviews of Interventions 2025) and the preferred reporting items for systematic reviews and meta‐analysis (PRISMA) framework (Page et al. 2021). These frameworks guided the formulation of the study design, the systematic and stepwise execution of the process, the statistical analysis, and the presentation of results. The study protocol was registered in the international prospective register of systematic reviews (PROSPERO) under registration number CRD420251128250.

### 2.2 Search Strategy and Databases

A systematic electronic search of PubMed, Embase, and Scopus was performed, encompassing all available records from database inception to July 2025, without any language restrictions. The following keywords were utilized: “PCA infarction”, “EVT”, “mechanical thrombectomy”, “PCA stroke”. A comprehensive search strategy is available in the Supplementary Table 1.

### 2.3 Study Selection and Eligibility Criteria

All studies identified through the electronic search were imported into Rayyan software for systematic screening, and duplicate entries were removed. The remaining studies underwent initial screening based on titles and abstracts. Full‐text articles were retrieved for detailed evaluation if deemed potentially relevant by either reviewer. Two independent reviewers (S.F and U.A) assessed the eligibility of each study in accordance with predefined inclusion criteria. Any disagreement in judgment was resolved through discussion, with arbitration by a third reviewer (S.R).

Studies were included if they met the following criteria: (1) involved patients suffering from PCA stroke; (2) endovascular treatment as intervention; (3) BMM as control; and (4) reported at least one relevant outcome.

Exclusion criteria included: (1) overlapping populations, defined by shared institutions and recruitment periods. To ensure dataset independence and avoid duplication, all eligible studies were carefully reviewed for potential overlapping patient populations. We cross‐checked study identifiers, including institution names, registry sources, and recruitment timelines. When two or more studies were part of the same collaborative network or registry (for example, Nguyen et al., 2023 and Strambo et al., 2024, both from the PLATO collaboration), we verified through each paper's inclusion period and patient selection criteria that there was no overlap of participants. In all such cases, datasets were confirmed to be independent before inclusion in the quantitative synthesis; (2) populations outside the scope of interest; (3) republished literature; (4) protocols without reported results; (5) reviews, abstracts, case reports, case series, background articles, expert opinions, or in vivo/in vitro studies; (6) duplicate data from the same clinical trial; and (7) absence of a comparator group.

### 2.4 Data Extraction and Outcomes

Two authors (A.M., M.F.) extracted data from the included studies into an Excel sheet using a pre‐piloted form. Baseline data included country, sample size, age, female, NIHSS at admission, IVT, smoking, hypertension, diabetes, atrial fibrillation, previous stroke, Hyperlipidemia, coronary heart disease, and anticoagulant use. Outcomes were categorized into primary and secondary outcomes. The primary outcomes were mRS scores of 0–2 at 90 days and mRS score of 0–1 at 90 days. The secondary outcomes were visual field normalization, ENI, symptomatic ICH, mortality rate at 90 days, and change in NIHSS at 24 h.

### 2.5 Quality Assessment

The quality of observational studies was assessed using the Newcastle‐Ottawa Quality assessment form for observational studies (Ottawa Hospital Research Institute 2025), which includes three domains: selection, comparability, and outcome. Thresholds were applied to convert the Newcastle‐Ottawa scores to AHRQ standards (good, fair, and poor). Two reviewers independently performed the assessment (U.A., F.Z.), with disagreements resolved through discussion or consultation with a third reviewer (S.R.). The selection domain was rated with a maximum of four stars, the comparability domain with a maximum of two stars, and the outcome domain with a maximum of three stars. Studies scoring 7–9 stars were rated as “low risk of bias,” studies scoring 5–6 stars were rated as “some concerns,” and studies scoring less than five stars were rated as “high risk of bias,” ensuring a comprehensive evaluation. This tool was also utilized in a previous study (Alsabri et al. 2025), ensuring methodological rigor.

### 2.6 Grade Assessment

The grading of recommendations, assessment, development, and evaluation (GRADE) tool was employed by two independent authors (S.R U.A) using the GRADEpro guideline development tool (Antesso et al. 2016) to evaluate the level of certainty of the evidence in this meta‐analysis, with categorizations ranging from high to very low (Shao et al. 2023). This approach was also utilized in our previous study, ensuring consistency in the appraisal of evidence quality (Rath et al. 2025). Any disagreements were discussed and resolved through consensus.

### 2.7 Statistical Analysis and Sensitivity Analysis

Statistical analyses were performed using R software and R Studio (version 4.4.2; R Core Team, Vienna, Austria, 2024.10.31 + 87279). The inverse variance method was used to calculate pooled effect estimates with corresponding 95% CIs (Fleiss 1993). A random‐effects model was applied when substantial heterogeneity was detected, whereas a fixed‐effects model was used for homogeneous outcomes. Dichotomous outcomes were expressed as ORs with 95% CIs, and results were graphically presented using forest plots.

Between‐study heterogeneity was assessed using the restricted maximum likelihood (REML) method to estimate the between‐study variance (τ^2^). The Q‐profile method was employed to compute CIs for both τ^2^ and τ (Viechtbauer 2005). Heterogeneity was quantified using the *I^2^* statistic, interpreted as follows: 0%–25% (low), 25%–50% (moderate), and >50% (substantial heterogeneity). To enhance interpretability, prediction intervals were also calculated where applicable, reflecting the expected range of effects in future comparable studies.

To evaluate the robustness of the pooled estimates, influence diagnostics and leave‐one‐out sensitivity analyses were conducted. Stratified (subgroup) forest plots were generated to visually examine sources of heterogeneity and assess consistency across subgroups.

Potential publication bias was explored using contour‐enhanced funnel plots, which distinguish areas of statistical significance and integrate study‐level power analysis to assess whether asymmetry may result from small, underpowered studies. These plots also report the median power, the minimum true effect size needed for 33% and 66% power, the test for excess significance (Ioannidis and Trikalinos 2007), and the replicability index (A Revised Introduction to the R‐Index). Funnel plot symmetry was further tested using Egger's regression test (Egger et al. 1997).

Overall, this comprehensive analytic framework—combining inverse variance weighting, REML‐based heterogeneity estimation, sensitivity diagnostics, and bias assessment—ensures both the statistical rigor and transparency of the pooled results.

## 3 Results

### 3.1 Screening and Study Selection

**FIGURE 1:**
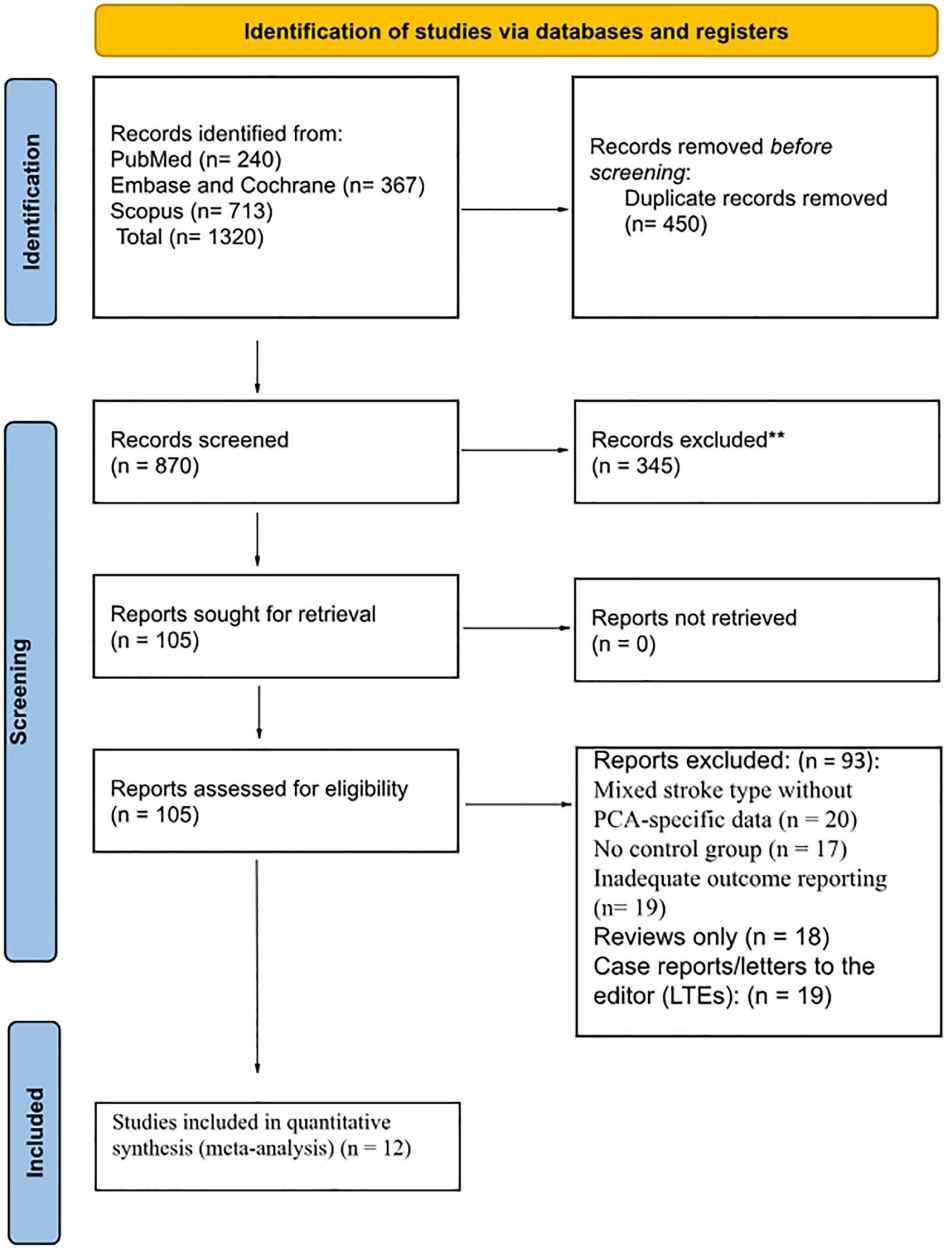
PRISMA flowchart of included studies.

The search identified 1320 records: 367 from Embase, 240from PubMed, and 713 from Scopus. After removing 450 duplicate records, 870 studies remained for the title and abstract screening. Following the screening process, 765 studies were excluded based on title and abstract evaluation. 105 full‐text articles were assessed for eligibility; 12 studies were included in the qualitative and quantitative synthesis. These comprised observational studies (Kalyoncu Aslan et al. 2025; Cunha et al. 2022; Dicpinigaitis et al. 2024; Herweh et al. 2021; Maulucci et al. 2023; Meyer et al. 2021; Mohammaden et al. 2024; Nguyen et al. 2023; Räty et al. 2024; Sabben et al. 2023; Salim et al. 2025; Strambo et al. 2024). The PRISMA flow diagram is given in Figure 1.

### 3.2 Study Characteristics

A total of 39,525 patients were included across the analyzed studies, with 2540 patients in the EVT group and 37075 in the BMM group. The studies were published between 2021 and 2025. The detailed baseline characteristics are presented in Table 1.

**TABLE 1:** Baseline Characteristics of included studies

Study ID	Country	Study Design	Sample Size	Age, mean (SD)		Female n (%)		NIHSS at Admission mean (SD)		IVT, n (%)		Smoking n (%)		Hypertension n (%)		Diabetes n (%)		Atrial fibrillation n (%)		Previous Stroke n (%)		Hyperlipidemia n (%)		Coronary heart disease n (%)		Anticoagulants use n (%)	
				EVT	BMM	EVT	BMM	EVT	BMM	EVT	BMM	EVT	BMM	EVT	BMM	EVT	BMM	EVT	BMM	EVT	BMM	EVT	BMM	EVT	BMM	EVT	BMM
Mohammaden 2024	USA & Europe	Retrospective cohort	321.0	69.4 ± 13	66.4±13.5	118 (65.9)	84 (59.2)	10 (Baseline score)	6	74 (41.3)	58 (40.8)	42 (23.5)	37 (26.1)	145 (81)	108 (76.1)	60 (33.5)	56 (39.4)	56 (31.3)	35 (24.6)	30 (16.8)	16 (11.3)	67 (37.4)	59 (41.5)	17 (9.5)	12 (8.5)	74 (41.3)	58 (40.8)
Herweh 2021	Germany, USA, Taiwan	Retrospective cohort	130.0	70 ± 13.3	74 ± 13.1	9 (39.1)	51 (47.7)	10 ± 15.01	15.33 ± 2.80	5 (21.7)	44 (41.1)	5 (20)	17 (15.9)	16 (69.6)	97 (90.7)	4 (17.4)	48 (44.9)	6 (26.1)	41 (38.7)	6 (26.1)	29 (27.1)	8 (36.4)	72 (67.3)	8 (34.8)	26 (24.3)	4 (17.5)	24 (22.6)
Sabben 2023	France, Switzerland, USA	multicenter retrospective	752.0	72 ± 14.96	73.3 (14.86)	79 (47.3)	250 (42.7)	8 ± 4.49	6.33 ± 5.20	90(53.9)	467(79.8)	30(18.0)	98 (16.9)	108 (64.7)	377 (64.4)	30 (18.0)	99 (16.9)	43(25.9)	118 (20.2)	16 (9.8)	89 (15.3)	NR	NR	NR	NR	21(12.6)	49(8.4)
Strambo 2024	Switzerland	multicenter, retrospective, case‐control	1059.0	72.4 ± 13.6	73.6 ± 11.2	159 (43.7)	293 (42.2)	8.33 ± 5.21	6 ± 5.2	145 (39.8)	292 (42.0)	46 (14.8)	141 (21.0)	275 (75.6)	541 (77.8)	87 (23.9)	207 (29.8)	114 (31.3)	193 (27.8)	46 (13.1)	145 (20.9)	153 (42.2)	368 (53.0)	NR	NR	60 (17.3)	92 (13.3)
Salim 2024	USA	Retrospective, multicenter	177.0	72.33 ± 15.8	67.67 ± 11.31	32 (36.0)	28 (26.0)	9.33 ± 6.03	6.60 ± 4.67	41(45%)	55(63)	15(17)	14(16)	58(65)	63(72)	19(21)	35(40)	20(22)	23(26)	NR	NR	38(43)	52(59)	NR	NR	10(11)	6(9.1)
Raty 2024	Europe, North America, Asia	multicenter retrospective	724.0	73.3 ± 13.39	74±12.99	172 (43)	134 (41.4)	8.33 ± 5.21	6.33±5.21	172 (43.0)	134 (41.4)	51 (15.0)	53(17.3)	305 (76.3)	234 (72.2)	92 (23.0)	74 (22.8)	131 (32.8)	88 (27.2)	50 (12.9)	56 (17.3)	171 (42.9)	159 (49.2)	64 (16.17)	58 (17.9)	64 (16.8)	23(7.2)
Nguyen 2023	Europe, North America	multinational case‐control	1023.0	73.33 ± 13.40	73.67 ± 13.37	162 (42.9)	272 (42.2)	8.33 ± 5.21	5.33 ± 5.20	152 (40.2)	287 (45.5)	48(15.1)	122(19.8)	288 (76.2)	497 (77.1)	86 (22.8)	191 (29.6)	126 (33.3)	185 (28.7)	47 (12.8)	146 (22.8)	161 (42.7)	346 (53.7)	NR	NR	63 (17.5)	91 (14.2)
Cunha 2022	Portugal	Retrospective, single‐center	38.0	71.4 ± 14.4	73.4 ± 14.1	10 (50.9)	17 (56.7)	6.7 ± 4.1	5.6 ± 4.1	4 (20.0%)	6 (20.0)	9 (45.0)	12 (40.0)	14 (70.0)	21 (70.0)	4 (20.0)	6 (20.0)	3 (15.0)	7 (23.3)	2 (10.0)	4 (13.3)	9 (45.0)	13 (43.3)	2 (10.0)	3 (10.0)	4 (20.0)	6 (20.0%)
Meyer 2021	Europe, United States, Asia	multicenter case‐control	243.0	72.3 ± 14.31	72.3 ± 14.31	45 (48.9)	50 (54.3)	6.17 ± 5.27	5.67± 6.03	37(40.2)	53 (57.6)	NR	NR	70 (76.1)	74(80.4%)	4 (15.2)	30 (32.6%)	33 (35.9)	33 (35.9%)	NR	NR	35 (38.0)	43 (46.7)	NR	NR	NR	NR
Maulucci 2023	USA	multicenter retrospective	268.0	72.2 ± 9.7	75.7 ± 8.6	42 (35.3)	68 (45.6)	9.3 ± 5.1	6.1 ± 4.3	69 (58.0)	149 (100.0)	NR	NR	78 (65.5)	99 (66.4)	21 (17.6)	29 (19.5)	28 (23.5)	36 (24.2)	10 (8.4)	12 (8.1)	37 (31.1)	45 (30.2)	11 (9.2)	15 (10.1)	15 (12.6)	6 (4.0)
Aslan 2025																											
	Turkey	Retrospective, single‐center	69.0	76 ± 11.89	73.72 ± 12.99	10 (62.5)	25 (47.2)	10.75 ± 4.93	6.64 ± 4.33	NR	NR	5 (31.3)	27 (50.9)	14 (87.5)	44 (83.0)	7 (43.8)	21 (39.6)	7 (43.8)	19 (35.8)	1 (6.3)	11 (20.8)	5 (31.3)	14 (26.4)	3 (18.8)	10 (18.9)	2 (12.5)	6 (11.3)
Dicpingaitis 2024	USA	Retrospective multicenter database (NIS)	34880.0	72 ± 13.16	71 ± 12.33	275 (37.7)	15,905 (46.6)	11.3 ± 6.7	3.3 ± 2.8	240 (32.9)	3785 (11.1)	235 (32.2)	13,060 (38.2)	635 (87.0)	29,870 (87.5)	260 (35.6)	13,505 (39.5)	305 (41.8)	9825 (28.8)	50 (6.8)	3105 (9.1)	450 (61.6)	22,035 (64.5)	65 (8.9)	2955 (8.7)	NR	NR

Demographics and Clinical Presentation											
Study ID	Country	type of study	sample size	Age, mean (SD)		Female n (%)		NIHSS at Admission mean (SD)		IVT, n (%)	
				EVT	BMM	EVT	BMM	EVT	BMM	EVT	BMM
Mohammaden 2024	USA & Europe	Retrospective cohort	321.0	69.4 ± 13	66.4±13.5	118 (65.9)	84 (59.2)	10 (Baseline score)	6	74 (41.3)	58 (40.8)
Herweh 2021	Germany, USA, Taiwan	Retrospective cohort	130.0	70 ± 13.3	74 ± 13.1	9 (39.1)	51 (47.7)	10 ± 15.01	15.33 ± 2.80	5 (21.7)	44 (41.1)
Sabben 2023	France, Switzerland, USA	multicenter retrospective	752.0	72 ± 14.96	73.3 (14.86)	79 (47.3)	250 (42.7)	8 ± 4.49	6.33 ± 5.20	90(53.9)	467(79.8)
Strambo 2024	Switzerland	multicenter, retrospective, case‐control	1059.0	72.4 ± 13.6	73.6 ± 11.2	159 (43.7)	293 (42.2)	8.33 ± 5.21	6 ± 5.2	145 (39.8)	292 (42.0)
Salim 2024	USA	Retrospective, multicenter	177.0	72.33 ± 15.8	67.67 ± 11.31	32 (36.0)	28 (26.0)	9.33 ± 6.03	6.60 ± 4.67	41(45%)	55(63)
Raty 2024	Europe, North America, Asia	multicenter retrospective	724.0	73.3 ± 13.39	74±12.99	172 (43)	134 (41.4)	8.33 ± 5.21	6.33±5.21	172 (43.0)	134 (41.4)
Nguyen 2023	Europe, North America	multinational case‐control	1023.0	73.33 ± 13.40	73.67 ± 13.37	162 (42.9)	272 (42.2)	8.33 ± 5.21	5.33 ± 5.20	152 (40.2)	287 (45.5)
Cunha 2022	Portugal	Retrospective, single‐center	38.0	71.4 ± 14.4	73.4 ± 14.1	10 (50.9)	17 (56.7)	6.7 ± 4.1	5.6 ± 4.1	4 (20.0%)	6 (20.0)
Meyer 2021	Europe, United States, Asia	multicenter case‐control	243.0	72.3 ± 14.31	72.3 ± 14.31	45 (48.9)	50 (54.3)	6.17 ± 5.27	5.67± 6.03	37(40.2)	53 (57.6)
Maulucci 2023	USA	multicenter retrospective	268.0	72.2 ± 9.7	75.7 ± 8.6	42 (35.3)	68 (45.6)	9.3 ± 5.1	6.1 ± 4.3	69 (58.0)	149 (100.0)
Aslan 2025											
	Turkey	Retrospective, single‐center	69.0	76 ± 11.89	73.72 ± 12.99	10 (62.5)	25 (47.2)	10.75 ± 4.93	6.64 ± 4.33	NR	NR
Dicpingaitis 2024	USA	Retrospective multicenter database (NIS)	34880.0	72 ± 13.16	71 ± 12.33	275 (37.7)	15,905 (46.6)	11.3 ± 6.7	3.3 ± 2.8	240 (32.9)	3785 (11.1)

Vascular Risk Factors																
Study ID	Smoking n (%)		Hypertension n (%)		Diabetes n (%)		Atrial fibrillation n (%)		Previous Stroke n (%)		Hyperlipidemia n (%)		Coronary heart disease n (%)		Anticoagulants use n (%)	
	**EVT**	**BMM**	**EVT**	**BMM**	**EVT**	**BMM**	**EVT**	**BMM**	**EVT**	**BMM**	**EVT**	**BMM**	**EVT**	**BMM**	**EVT**	**BMM**
Mohammaden 2024	42 (23.5)	37 (26.1)	145 (81)	108 (76.1)	60 (33.5)	56 (39.4)	56 (31.3)	35 (24.6)	30 (16.8)	16 (11.3)	67 (37.4)	59 (41.5)	17 (9.5)	12 (8.5)	74 (41.3)	58 (40.8)
Herweh 2021	5 (20)	17 (15.9)	16 (69.6)	97 (90.7)	4 (17.4)	48 (44.9)	6 (26.1)	41 (38.7)	6 (26.1)	29 (27.1)	8 (36.4)	72 (67.3)	8 (34.8)	26 (24.3)	4 (17.5)	24 (22.6)
Sabben 2023	30(18.0)	98 (16.9)	108 (64.7)	377 (64.4)	30 (18.0)	99 (16.9)	43(25.9)	118 (20.2)	16 (9.8)	89 (15.3)	NR	NR	NR	NR	21(12.6)	49(8.4)
Strambo 2024	46 (14.8)	141 (21.0)	275 (75.6)	541 (77.8)	87 (23.9)	207 (29.8)	114 (31.3)	193 (27.8)	46 (13.1)	145 (20.9)	153 (42.2)	368 (53.0)	NR	NR	60 (17.3)	92 (13.3)
Salim 2024	15(17)	14(16)	58(65)	63(72)	19(21)	35(40)	20(22)	23(26)	NR	NR	38(43)	52(59)	NR	NR	10(11)	6(9.1)
Raty 2024	51 (15.0)	53(17.3)	305 (76.3)	234 (72.2)	92 (23.0)	74 (22.8)	131 (32.8)	88 (27.2)	50 (12.9)	56 (17.3)	171 (42.9)	159 (49.2)	64 (16.17)	58 (17.9)	64 (16.8)	23(7.2)
Nguyen 2023	48(15.1)	122(19.8)	288 (76.2)	497 (77.1)	86 (22.8)	191 (29.6)	126 (33.3)	185 (28.7)	47 (12.8)	146 (22.8)	161 (42.7)	346 (53.7)	NR	NR	63 (17.5)	91 (14.2)
Cunha 2022	9 (45.0)	12 (40.0)	14 (70.0)	21 (70.0)	4 (20.0)	6 (20.0)	3 (15.0)	7 (23.3)	2 (10.0)	4 (13.3)	9 (45.0)	13 (43.3)	2 (10.0)	3 (10.0)	4 (20.0)	6 (20.0%)
Meyer 2021	NR	NR	70 (76.1)	74 (80.4%)	4 (15.2)	30 (32.6%)	33 (35.9)	33 (35.9%)	NR	NR	35 (38.0)	43 (46.7)	NR	NR	NR	NR
Maulucci 2023	NR	NR	78 (65.5)	99 (66.4)	21 (17.6)	29 (19.5)	28 (23.5)	36 (24.2)	10 (8.4)	12 (8.1)	37 (31.1)	45 (30.2)	11 (9.2)	15 (10.1)	15 (12.6)	6 (4.0)
Aslan 2025	5 (31.3)	27 (50.9)	14 (87.5)	44 (83.0)	7 (43.8)	21 (39.6)	7 (43.8)	19 (35.8)	1 (6.3)	11 (20.8)	5 (31.3)	14 (26.4)	3 (18.8)	10 (18.9)	2 (12.5)	6 (11.3)
Dicpingaitis 2024	235 (32.2)	13,060 (38.2)	635 (87.0)	29,870 (87.5)	260 (35.6)	13,505 (39.5)	305 (41.8)	9825 (28.8)	50 (6.8)	3105 (9.1)	450 (61.6)	22,035 (64.5)	65 (8.9)	2955 (8.7)	NR	NR

**Abbreviations**: EVT, endovascular therapy; BMM, best medical management; NIHSS, National Institutes of Health Stroke Scale; IVT, intravenous thrombolysis; SD, standard deviation; n (%), number (percentage); NR, not reported; USA, United States of America; NIS, National Inpatient Sample.

### 3.3 Risk of Bias Assessment

The quality assessment through the Newcastle Ottawa Scale classified the studies as having low risk with scores ranging from 7 to 9. Detailed assessments is presented in Supplementary Table 2.

### 3.4 Grade Assessment

The GRADE assessment demonstrated that endovascular therapy (EVT) provides significant clinical benefits over best medical therapy (BMT) in patients with PCA stroke. EVT was associated with higher rates of functional independence (mRS 0–2 at 90 days) and excellent outcomes (mRS 0–1), with moderate certainty of evidence and upgrades for large treatment effects. It also conferred reductions in all‐cause mortality and consistent improvements in neurological outcomes, including greater odds of early neurological recovery and an average 1.3‐point improvement in NIHSS scores, again with moderate certainty. Importantly, the risk of symptomatic intracranial hemorrhage did not differ meaningfully between EVT and BMT, indicating a neutral safety profile. However, evidence regarding visual field outcomes was very limited, inconsistent, and imprecise, resulting in very low certainty. Overall, EVT demonstrates moderate‐certainty evidence of improved survival and functional recovery without a major increase in safety risks, although further high‐quality studies are needed to clarify its effects on visual outcomes. Detailed assessments is presented in Table 2.

**TABLE 2:** GRADE summary of findings table.

Outcome	Studies (participants)	Pooled effect (95% CI)	Absolute risk (per 1000)	Certainty (GRADE)	Comments
Functional independence (mRS 0–2 at 90d)	12 studies (*N* = 4352)	OR 1.86 (1.47–2.35), *I^2^* = 39%	BMT: 320 → EVT: 452	⨁⨁⨁◯ moderate	Large effect, low‐moderate heterogeneity.
Excellent outcome (mRS 0–1)	11 studies (*N* = 4091)	OR 2.32 (1.66–3.23), *I^2^* = 44%	BMT: 150 → EVT: 281	⨁⨁⨁◯ moderate	Upgraded for magnitude of benefit.
All‐cause mortality	11 studies (*N* = 4091)	OR 0.76 (0.69–0.84), *I^2^* = 23%	BMT: 180 → EVT: 137	⨁⨁⨁◯ moderate	Consistent mortality reduction.
Early neurological improvement	7 studies (*N* = 3040)	OR 2.11 (1.81–2.45), *I^2^* = 0%	BMT: 250 → EVT: 444	⨁⨁⨁◯ moderate	No heterogeneity, upgraded for large effect.
Change in NIHSS	6 studies (*N* = 1747)	MD –1.30 (–1.89 to –0.71), *I^2^* = 0%	−1.3 point improvement	⨁⨁⨁◯ moderate	Precision and consistency support upgrade.
Symptomatic ICH (sICH)	12 studies (*N* = 4937)	OR 1.06 (0.92–1.22), *I^2^* = 0%	BMT: 40 → EVT: 42	⨁⨁⨁◯ moderate	Neutral effect, consistent and precise.
Visual field deficit	3 studies (*N* = 379)	OR 1.23 (0.23–6.48), *I^2^* = 88%	BMT: 150 → EVT: 185	⨁◯◯◯ Very low	Sparse data, high inconsistency & imprecision.

Comparison: endovascular therapy (EVT) vs best medical management (BMT) in posterior cerebral artery (PCA) stroke.

Study design: observational studies.

Risk of bias: mostly low to moderate (NOS 8–9 stars).

### 3.5 Meta‐Analysis of Outcomes

#### 3.5.1 ENI

**FIGURE 2:**
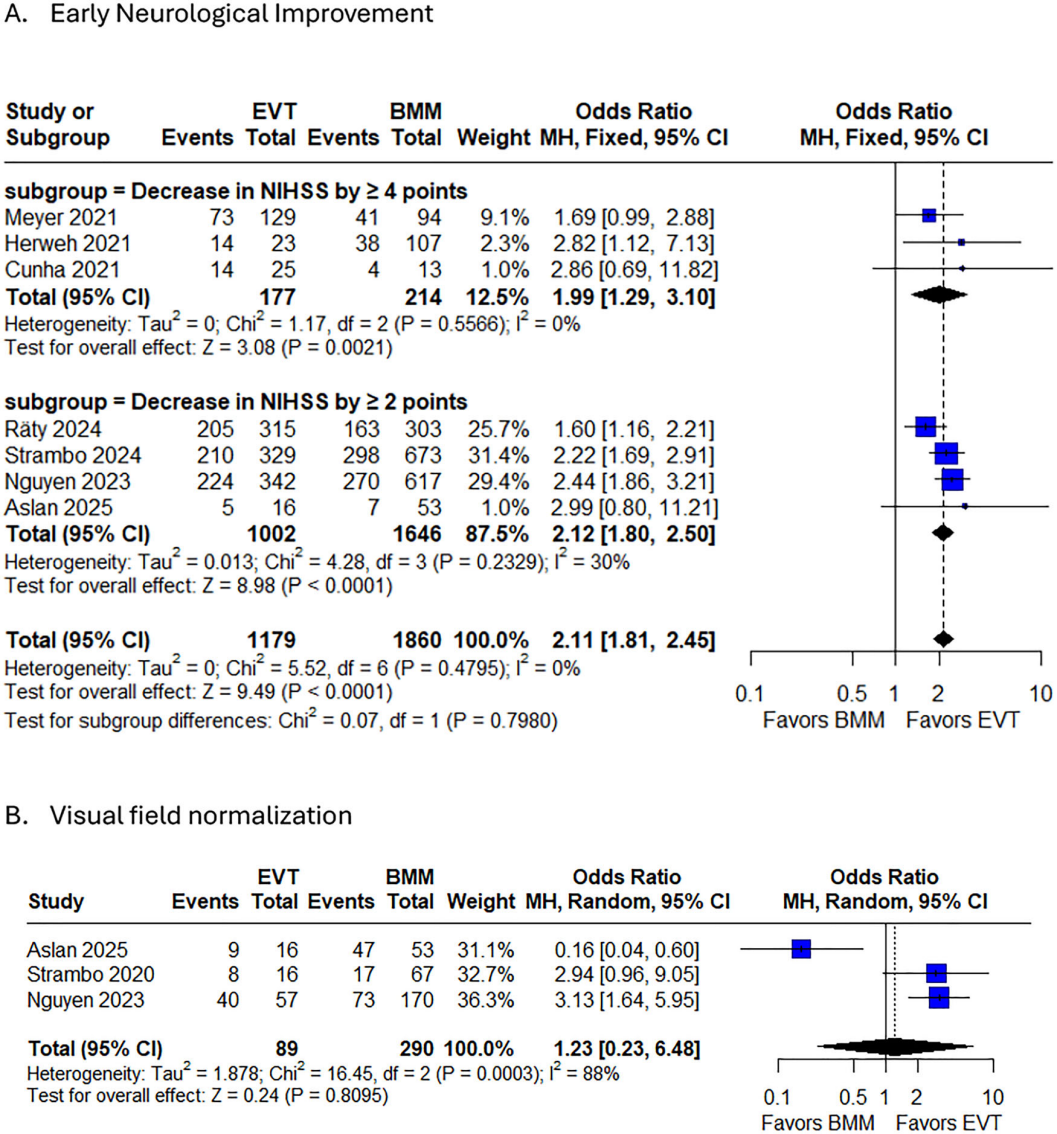
Forest plot of A. Early neurological outcome; B. Visual field normalization.

Pooled analysis demonstrated that EVT was associated with significantly higher odds of ENI, defined as a reduction in NIHSS score by ≥ 2 or ≥ 4 points, compared with BMM (OR 2.11, 95% CI 1.81–2.45, *p* < 0.0001; *I^2^* = 0%). Subgroup analyses revealed consistent benefits, with an OR of 1.99 (95% CI 1.29–3.10, *p* = 0.002; *I^2^* = 0%) for NIHSS improvement ≥ 4 points and an OR of 2.12 (95% CI 1.80–2.50, *p* < 0.0001; *I^2^* = 30%) for improvement ≥ 2 points, with no significant subgroup differences (*p* = 0.80) (Figure 2A). Sensitivity analyses, in which individual studies were sequentially omitted, confirmed the robustness of these findings, with ORs ranging from 1.97 to 2.28 and minimal heterogeneity (*I^2^* ≤ 6%) (Supplementary Figure 1). Additionally, funnel plot inspection revealed no evidence of publication bias (Supplementary Figure 2). These results suggest that EVT significantly improves early neurological recovery in PCA stroke compared with medical management alone, with consistent effects across varying definitions of ENI.

#### 3.5.2 Visual Field Normalisation

Pooled analysis of visual field outcomes demonstrated no significant difference between EVT and BMM (OR 1.23, 95% CI 0.23–6.48, *p* = 0.8095), though substantial heterogeneity was observed (*I^2^* = 88%, Tau^2^ = 1.878) (Figure 2B). The forest plot revealed marked variability across studies: while Nguyen 2023 showed significantly improved visual fields with EVT (OR 3.13, 95% CI 1.64–5.95), Asian 2025 paradoxically favored BMM (OR 0.16, 95% CI 0.04–0.60). Sensitivity analyses highlighted the instability of these findings, excluding Asian 2025 increased the point estimate to 3.08 (95% CI 1.76–5.38) with resolved heterogeneity (*I^2^* = 0%), whereas omitting Nguyen 2023 or Strambo 2020 rendered the effect nonsignificant (OR 0.71–0.76) with persistent heterogeneity (*I^2^* ≥ 91%) (Supplementary Figure 3). Funnel plot asymmetry suggested potential publication bias or methodological differences (Supplementary Figure 4).

#### 3.5.3 MRS 0–2 at 90 Days

**FIGURE 3:**
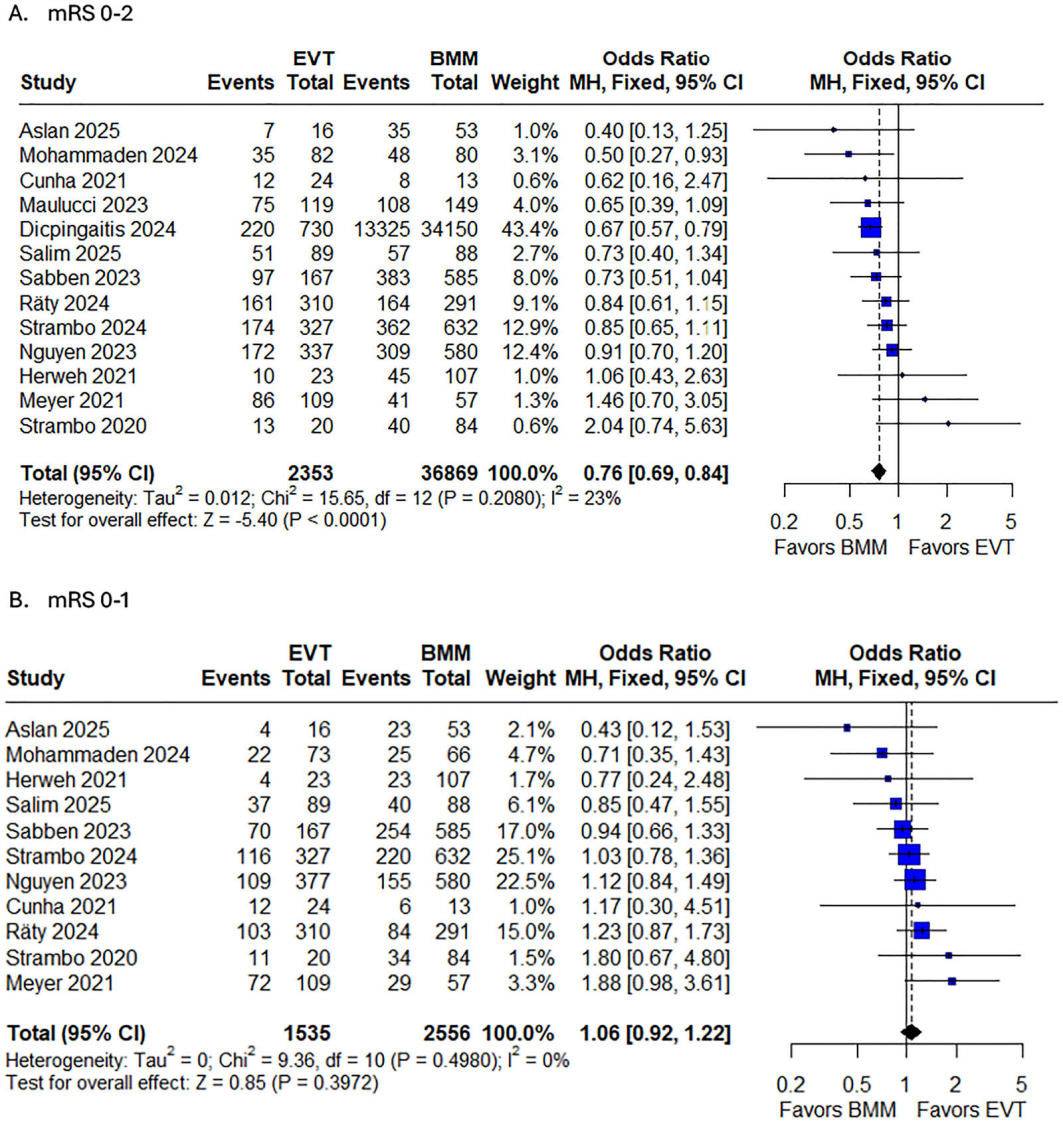
Forest plot of A. Mrs 0–2; B. MRS 0–1.

The meta‐analysis of functional outcomes (modified rankin scale (mRS) 0–2 at 90 days) revealed that EVT was associated with significantly reduced odds of achieving functional independence compared with BMM in PCA strokes (OR 0.75, 95% CI 0.67–0.85, *p* < 0.0001), with low heterogeneity (*I^2^* = 23%). While most studies (10/13) demonstrated consistent directionality favoring BMM, two smaller studies (Meyer 2021 and Strambo 2020) showed non‐significant trends toward EVT benefit. The pooled estimate was largely driven by the largest study (Dippingatits 2024), which contributed 43.4% of the weight (Figure 3A). Sensitivity analyses confirmed the robustness of these findings, as exclusion of any single study did not substantially alter the effect size or significance (Supplementary Figure 5). Funnel plot analysis suggested potential publication bias, with mild asymmetry indicating possible underrepresentation of small negative studies (Supplementary Figure 6).

#### 3.5.4 MRS 0–1 at 90 Days

The meta‐analysis of excellent functional outcomes (mRS 0–1) showed no significant difference between EVT and BMM (OR 1.06, 95% CI 0.92–1.22, *p* = 0.3972), with no observed heterogeneity (*I^2^* = 0%). The forest plot demonstrated a balanced distribution of effects across studies, with five studies favoring EVT (OR > 1.0) and six favoring BMM (OR < 1.0), though all CIs crossed unity. Notably, Meyer 2021 showed the strongest trend toward EVT benefit (OR 1.88, 95% CI 0.98‐3.61), while Asian 2025 suggested potential harm (OR 0.43, 95% CI 0.12–1.53) (Figure 3B). Sensitivity analyses confirmed the stability of these findings, as sequential exclusion of individual studies yielded nearly identical pooled estimates (OR range: 1.03–1.09) (Supplementary Figure 7). The symmetrical funnel plot indicated no evidence of publication bias (Supplementary Figure 8).

#### 3.5.5 Symptomatic ICH

**FIGURE 4:**
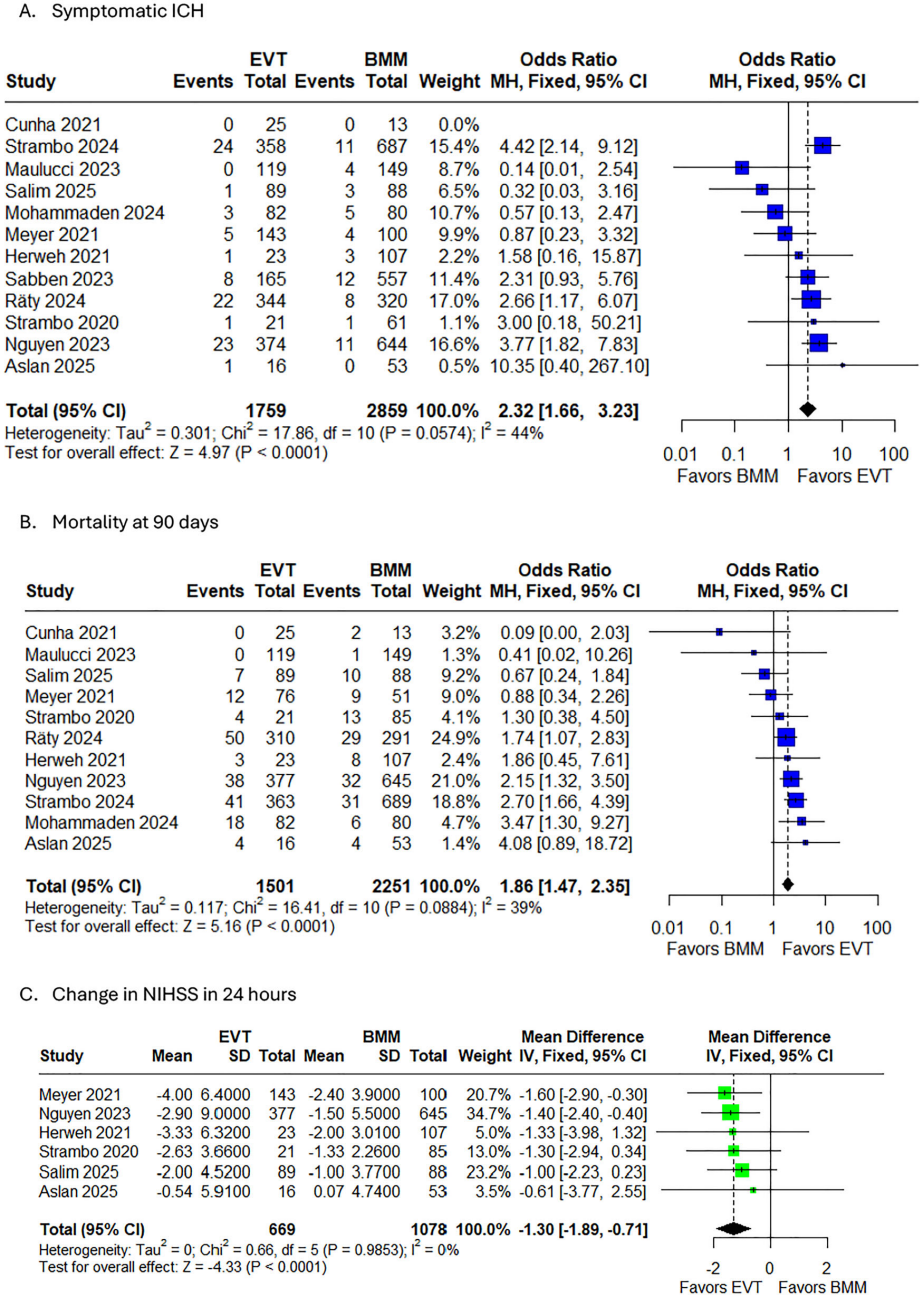
Forest plot of A. symptomatic ICH; B. Mortality at 90 days; C. Change in NIHSS at 24 h.

The meta‐analysis revealed a significantly increased risk of symptomatic intracranial hemorrhage (sICH) with EVT compared to BMM in (PCA strokes (OR 2.32, 95% CI 1.66‐3.23, *p* < 0.0001). While moderate heterogeneity was observed (*I^2^* = 44%), the majority of included studies demonstrated consistent directionality favoring increased hemorrhage risk with EVT, with four studies reaching statistical significance (Figure 4A). The risk elevation remained robust across sensitivity analyses, with ORs ranging from 1.94 to 2.53 when individual studies were sequentially excluded (Supplementary Figure 9). Notably, the Strambo 2024, Räty 2024, and Nguyen 2023 studies showed particularly strong associations between EVT and sICH risk, with ORs exceeding 2.5.

#### 3.5.6 Mortality Rates at 90 Days

The meta‐analysis of 90‐day mortality outcomes revealed significantly higher mortality rates with EVT compared to BMM in PCA stroke patients (OR 1.86, 95% CI 1.47–2.35, *p* < 0.0001) (Figure 4B). This concerning finding persisted across sensitivity analyses (OR range 1.66–1.98) and showed moderate heterogeneity (*I^2^* = 39%). While most studies demonstrated increased mortality with EVT, four showed statistically significant effects, with ORs ranging from 1.74 to 3.47 (Supplementary Figure 10). The funnel plot's asymmetry raises concerns about potential publication bias, suggesting the true mortality risk may be even higher than reported (Supplementary Figure 11).

#### 3.5.7 Change in NIHSS at 24 H

The meta‐analysis demonstrated that EVT was associated with significantly greater ENI compared with BMM, as measured by the mean reduction in NIHSS scores at 24 h (mean difference –1.30 points, 95% CI –1.89 to –0.71, *p* < 0.0001). This finding showed remarkable consistency across all included studies, with no observed heterogeneity (*I^2^* = 0%, Tau^2^ = 0). The forest plot revealed that while the magnitude of benefit varied slightly among studies (–0.61 to –1.60 points), all point estimates favored EVT, with Meyer 2021 showing the largest treatment effect (–1.60 points, 95% CI –2.90 to –0.30) (Figure 4C). Sensitivity analyses confirmed the robustness of these results, as the exclusion of any single study did not substantially alter the pooled estimate (range: –1.23 to –1.40 points) (Supplementary Figure 12). The symmetrical funnel plot suggested no evidence of publication bias (Supplementary Figure 13).

## 4 Discussion

In patients who present with PCA strokes, this meta‐analysis offers a thorough synthesis of the available data contrasting EVT with BMM. Due to anatomical differences, unique symptomatology, and a lack of randomized data, EVT's role in PCA stroke is still unknown, despite its obvious benefits in anterior circulation strokes. With some early neurological benefits seen after EVT, the current findings show a complex therapeutic landscape; however, long‐term functional outcomes and safety endpoints present a more cautious picture.

The ENI linked to EVT was the most consistent finding across studies. Regardless of whether a 2‐ or 4‐point reduction threshold was applied, patients treated with EVT had a significantly higher chance of showing improvement in NIHSS scores within 24 h. The pooled mean decrease of 1.3 points on the NIHSS in comparison with BMM further demonstrated the magnitude of this early benefit. According to these results, reperfusion in PCA stroke may quickly restore perfusion to expressive areas, resulting in noticeable neurological recovery. This is consistent with earlier findings from single‐arm studies that showed that successful recanalization in PCA infarcts was followed by an early improvement in consciousness, vision, and cognitive function (Alawieh et al. 2019; Imam et al. 2024).

Notwithstanding this initial benefit, EVT did not show comparable favorability in the long‐term functional outcomes. A mRS score of 0 to 2 at 90 days was substantially less likely to be attained by patients receiving EVT, suggesting a decreased likelihood of functional independence. There was no discernible benefit to EVT, and rates of excellent recovery (mRS 0–1) were similar across groups. More investigation is necessary into this seeming discrepancy between short‐term enhancement and long‐term performance. PCA strokes frequently cause visual field deficits, alexia, memory impairment, and cortical blindness, which may not be sufficiently recorded by the mRS, in contrast to anterior circulation strokes, which are characterized primarily by hemiparesis and speech impairments. The burden of disability unique to PCA infarcts may be underestimated by functional measures that are heavily weighted towards motor outcomes (Mattle et al. 2011; Sangpetngam et al. 2022).

There was no discernible difference between EVT and BMM in visual field normalization, an outcome specifically related to PCA stroke. Studies evaluating this endpoint, however, showed significant heterogeneity. Inconsistent results were probably caused by variations in the definitions of recovery, perimetry techniques, and assessment timing. Some studies found little change after recanalization, while others reported a significant improvement in homonymous hemianopia. One of the biggest obstacles to assessing actual benefit in this area is the absence of consistent visual outcome measures across trials (Berberich et al. 2022; Chang et al. 2024). Given that visual field loss can severely impact quality of life despite low mRS scores, its omission from most stroke trials is a critical gap in current research methodology.

Safety results brought up serious issues. Compared with BMM, EVT was linked to a risk of symptomatic intracranial haemorrhage (sICH) that was more than double. Given the technical difficulties in reaching distal PCA branches, the thinner vessel walls, and the possibility of reperfusion injury in ischemic but already damaged tissue, this finding held across sensitivity analyses and is biologically believable. Previous observational studies have reported higher rates of hemorrhagic conversion after mechanical manipulation in posterior circulation stroke (Pirson et al. 2022; Goyal et al. 2016). Notably, this risk was not mitigated in studies using newer‐generation stent retrievers or aspiration techniques, suggesting that technology alone may not eliminate this hazard.

In addition to sICH, EVT was associated with significantly higher 90‐day mortality compared with BMM. This finding contrasts sharply with the mortality benefit consistently reported in anterior circulation stroke trials such as HERMES, MR CLEAN, and ESCAPE (Saver et al. 2015; Jovin et al. 2015). This discrepancy could be caused by a number of factors. Due to subtle or varying symptoms, PCA strokes frequently result in delayed diagnosis, which can cause late presentation past the therapeutic window. Additionally, the thalamus and brainstem are included in the proximal segments of the PCA territory, and infarction in these areas is linked to respiratory compromise, autonomic dysfunction, and impaired consciousness, all of which can exacerbate outcomes independent of recanalization (Sairanen et al. 2011; Adusumilli et al. 2024). In some cases, EVT in PCA occlusions may result in futile recanalization, where reopening the vessel fails to salvage functionally meaningful tissue and instead contributes to hemorrhagic complications.

Each patient needs a careful risk‐benefit assessment because the high clinical stakes of the added sICH and 90‐day mortality risks of EVT in PCA stroke dictate that these factors could potentially influence the risk of complications. Clinicians need to prioritize patient selection according to time of symptom onset, site of occlusion (proximal vs. distal PCA), volume of infarct, and pre‐existing comorbidities. The potential for adverse results, say, might outweigh the benefits of early neurological recovery in patients with late presentation or infarcts of crucial structures such as brainstem or thalamus. With the potential that visual and cognitive dysfunction can impact quality of life even in the presence of low (mRS scores, it is crucial that patient preferences and expectations are considered in shared decision‐making. These considerations underscore the need for a tailored approach that balances the added danger of bleeding and mortality against the possible benefits of early reperfusion.

Significant differences in outcome definitions for mRS, NIHSS, and visual field recovery among included studies were addressed in the meta‐analysis, which made it difficult to synthesize the findings. Different perimetry methods, recovery thresholds, and assessment timing caused heterogeneity in visual field recovery; these were addressed by pooling data using standardized effect measures whenever feasible and using subgroup analyses to examine variances. Although NIHSS results were more uniformly defined, sensitivity analyses were necessary to guarantee the robustness of the pooled mean decrease of 1.3 points because early improvement thresholds (such as 2‐ or 4‐point reductions) differed. While a score of 0–2 was frequently used for mRS, variability was introduced by variations in adjudication techniques and follow‐up duration. This was addressed using random‐effects modeling to account for interstudy differences. The large CIs in pooled estimates, especially for visual outcomes and long‐term functional independence, were probably caused by this heterogeneity, which may have diluted the observed effect sizes and made direct comparisons more difficult. These methodological issues show that in order to increase the accuracy and comparability of results, future PCA stroke studies must use standardized outcome measures.

Crucially, the majority of the studies in this meta‐analysis were observational, which entails bias and confounding risks. Clinical judgment was frequently used to choose patients for EVT, which could lead to selection bias, particularly if the patients had larger infarct volumes or more severe strokes. Even though some studies used multivariable adjustment and propensity score matching, residual confounding is still a concern. Additionally, there were significant differences in imaging selection criteria between studies. While some relied solely on clinical symptoms or perfusion imaging, others included patients based on CTA‐confirmed occlusion. The heterogeneity observed in multiple outcomes was probably influenced by this discrepancy (Nogueira et al. 2018; Kim and Kim 2014; Maus et al. 2021).

All things considered, these results imply that although EVT might provide ENI in PCA stroke, its advantages do not always translate into improved long‐term function and could result in more complications. The appropriateness of mRS as the only outcome measure in this population is called into question by the apparent discrepancy between early improvement and functional independence. Careful patient selection and personalized decision‐making are essential due to the elevated risks of bleeding and death. Given the variety of anatomy and clinical presentations involved, it is unlikely that a single EVT strategy can be used for all PCA occlusions.

## 5 Strengths and Limitations

This meta‐analysis has several strengths. It is the most comprehensive synthesis to date focused specifically on EVT in PCA stroke, incorporating a broad range of outcomes beyond just mRS. By evaluating ENI, visual outcomes, hemorrhagic complications, and mortality, this study offers a more nuanced understanding of the trade‐offs involved. Sensitivity analyses and subgroup evaluations were conducted to test the robustness of the findings, and heterogeneity was generally low for the primary outcomes of interest. Moreover, the inclusion of multiple definitions for early improvement and grade assessment allows for a broader applicability of results across clinical settings.

Nonetheless, several limitations must be acknowledged. Most included studies were retrospective cohorts with inherent limitations in design. The lack of randomization increases the risk of selection bias, and confounding by indication remains a possibility. Outcome definitions were not uniform across studies, particularly for visual field assessment and sICH, which may have contributed to heterogeneity. Furthermore, the small sample sizes in several studies may have limited the power to detect significant differences in some outcomes. Publication bias cannot be excluded, although a formal assessment was not possible due to the small number of studies per outcome.

## 6 Future Implications

There is a pressing need for randomized controlled trials dedicated specifically to PCA stroke, with stratification based on occlusion location (proximal versus distal), infarct volume, and symptom profile. Given the limitations of mRS in capturing visual and cognitive deficits, future studies should incorporate more granular outcome measures such as visual field perimetry, memory testing, and quality of life scales. Standardization in imaging criteria and procedural techniques would also enhance the comparability of results. Stratifying by occlusion site, including visual and cognitive outcomes alongside mRS, and applying standardized imaging or procedural protocols, could significantly improve future study design. Until such data become available, clinical decision‐making for EVT in PCA stroke should be highly individualized, considering not only occlusion location and stroke severity but also patient comorbidities and time to presentation.

## 7 Conclusion

This meta‐analysis demonstrates that EVT in PCA stroke is associated with ENI but does not confer clear long‐term functional benefit and is linked to higher risks of symptomatic hemorrhage and mortality. These findings underscore the need for cautious, individualized use of EVT in this population and highlight the limitations of current outcome measures in capturing the true impact of PCA stroke. Future randomized trials with PCA‐specific endpoints are urgently needed to guide practice.

## Author Contributions

Conceptualization: Shree Rath and Umama Alam. Methodology: Shree Rath and Umama Alam. Investigation: Shree Rath and Umama Alam. Formal analysis: shree rath. Writing – original draft: Shree Rath, Umama Alam, Rabia Ahmed, Fazia Khattak, Muhammad Asad Asif, Alatisemonsurah Bisola, Lubaba Yunas, Adil Khan, Mohd Sijad Uddin, Labannya Das Puja, Sayed Inamullah, Mahin Fatima, Sumia Fatima, Fazeela Bibi and Abdul Moiz. Writing – review and editing: Shree Rath and Raheel Ahmed. Supervision: Raheel Ahmed.

## Funding

The authors have nothing to report.

## Conflicts of Interest

The authors declare no conflicts of interest.

## Ethics Statement

The authors have nothing to report.

## Supporting information



Supplementary Information

## Data Availability

Data is available in the manuscript and the supplementary file.
