# Supplementary material for: Endovascular Therapy Versus Best Medical Treatment in Posterior Cerebral Artery Stroke: A Systematic Review and Meta‐Analysis
Source: Brain Behav. 2026 Jan 7;16(1):e71194. doi: 10.1002/brb3.71194 (PMC12778430; doi:10.1002/brb3.71194)
Supplement: Supplementary file 1 — Supplementary Information [file BRB3-16-e71194-s001.docx]

Supplementary File:

Supplementary Table 1: Search strategy across included databases

| Database | String | Results |
| --- | --- | --- |
| PubMed | ("Thrombectomy"[MeSH Terms] OR "Endovascular Procedures"[MeSH Terms])  AND ("Posterior Cerebral Artery"[Title/Abstract] OR "posterior cerebral artery occlusion"[Title/Abstract]) | 240 |
| Embase | ('thrombectomy'/exp OR 'endovascular procedure'/exp)  AND ('posterior cerebral artery':ti,ab OR 'posterior cerebral artery occlusion':ti,ab) | 367 |
| Scopus | TITLE-ABS-KEY(thrombectomy OR "endovascular procedure*")  AND TITLE-ABS-KEY("posterior cerebral artery" OR "posterior cerebral artery occlusion") | 713 |

Supplementary Table 2: Quality assessment using Newcastle-Ottawa Scale

| STUDY ID | SELECTION | | | | COMPARIBILITY | | OUTCOMES | | | TOTAL |
| --- | --- | --- | --- | --- | --- | --- | --- | --- | --- | --- |
|  | S1 | S2 | S3 | S4 | C1 | C2 | O1 | O2 | O3 |  |
| Nguyen 2023 | * | * | * | — | * | * | — | * | * | 9 |
| Meyer 2021 | * | * | * | * | * | — | * | * | * | 8 |
| Mohammaden 2024 | * | * | * | * | * | — | * | * | * | 8 |
| Maulucci 2023 | * | * | * | — | * | * | * | * | * | 7 |
| Aslan 2025 | * | * | * | — | * | * | * | * | * | 8 |
| Dicpinigaitis 2024 | * | * | * | * | * | — | * | * | * | 8 |
| Strambo 2024 | * | * | * | * | * | * | — | * | * | 8 |
| Sabben 2023 | * | * | * | — | * | * | — | * | * | 7 |
| Salim 2024 | * | * | * | * | * | * | * | * | * | 9 |
| Raty 2024 | * | * | * | * | * | * | * | * | * | 9 |
| Cunha 2022 | * | * | * | * | * | * | * | * | * | 9 |
| Herweh 2021 | * | * | * | * | * | * | * | * | * | 9 |

Supplementary Figure 1: Sensitivity analysis for ENI


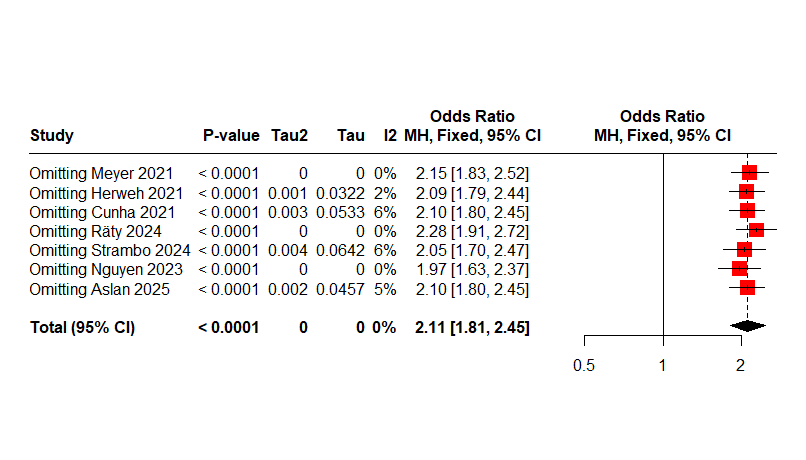


Supplementary Figure 2: Funnel plot for ENI


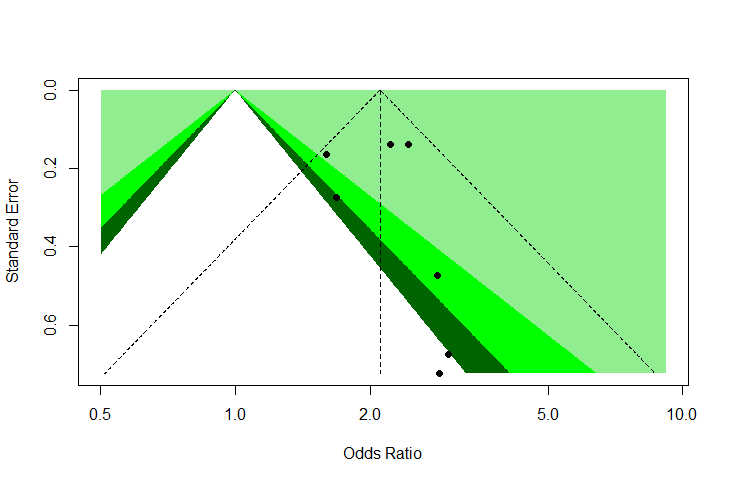


Supplementary Figure 3: Sensitivity analysis for visual field normalization


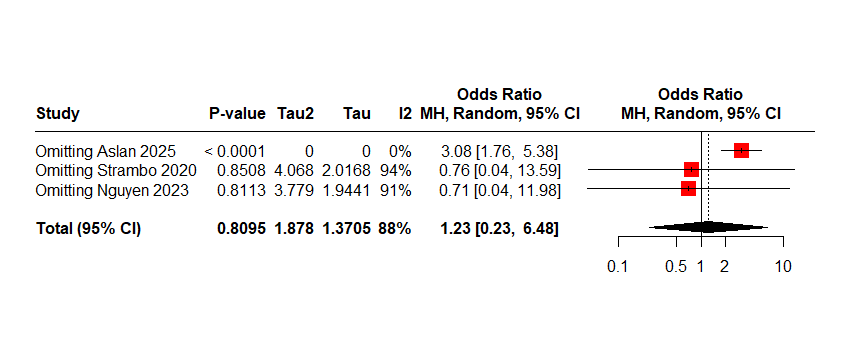


Supplementary Figure 4: Funnel plot for visual field normalization


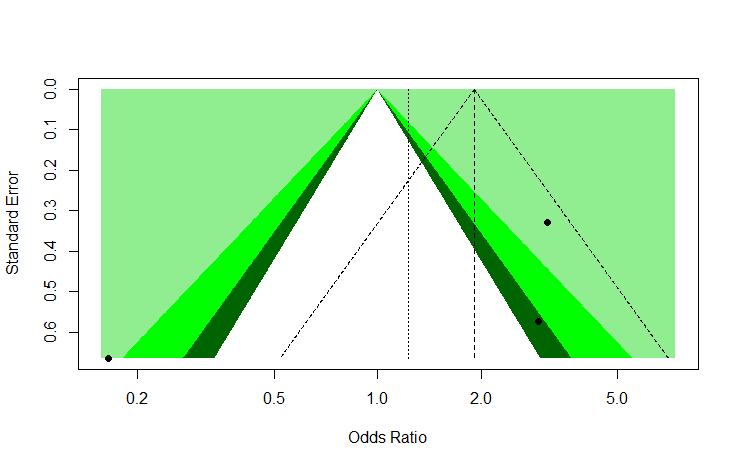


Supplementary Figure 5: Sensitivity analysis for Mrs 0-2


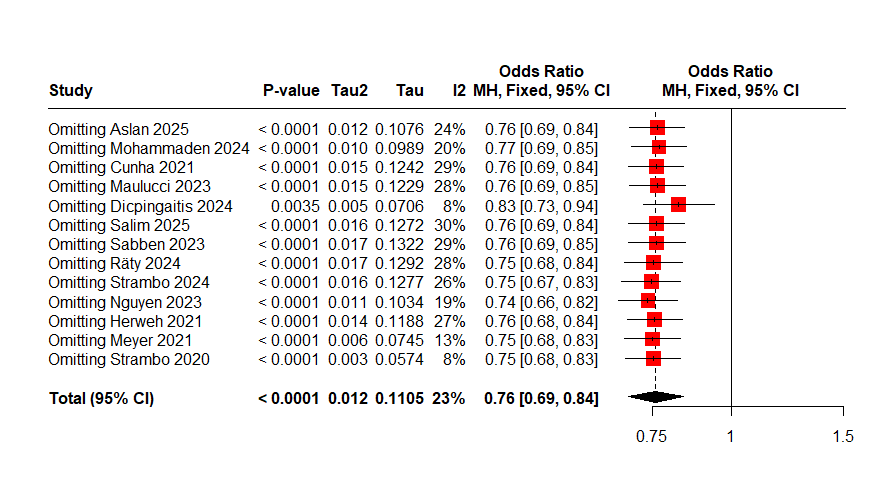


Supplementary Figure 6: Funnel plot for Mrs 0-2


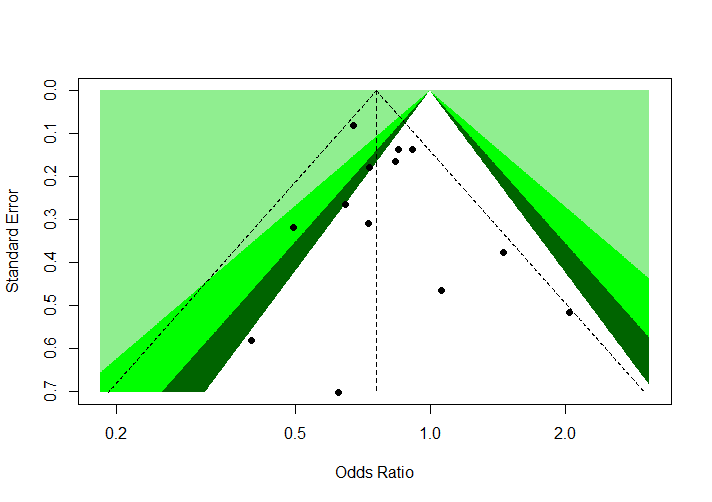


Supplementary Figure 7: Sensitivity analysis for mrs 0-1


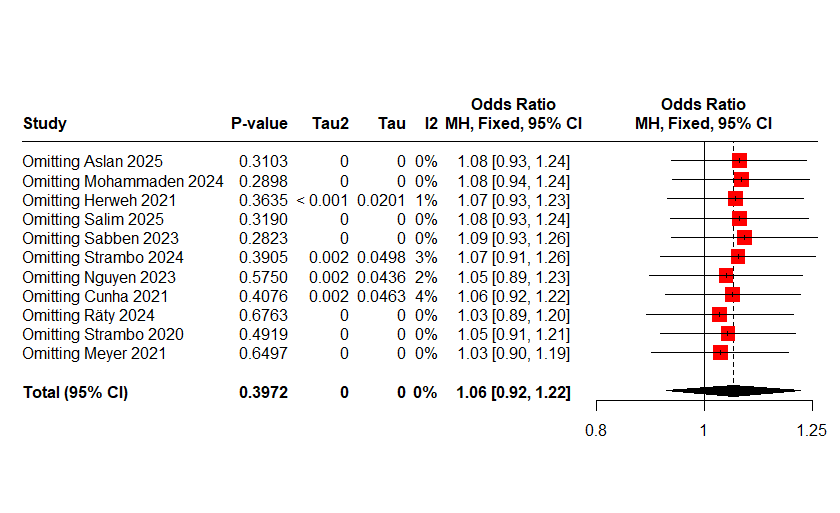


Supplementary Figure 8: Funnel plot for mrs 0-1


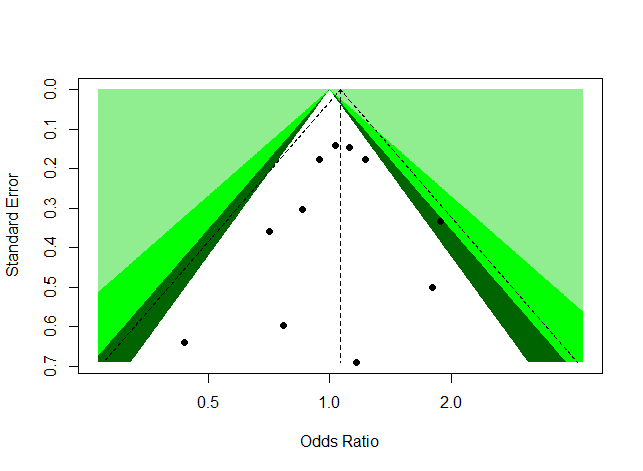


Supplementary Figure 9: Sensitivity analysis for symptomatic ICH


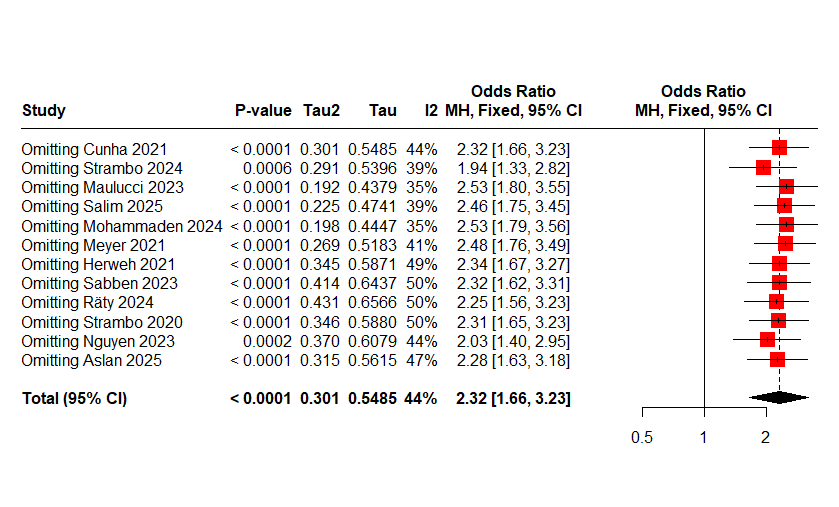


Supplementary Figure 10: Sensitivity analysis for mortality rates at 90 days


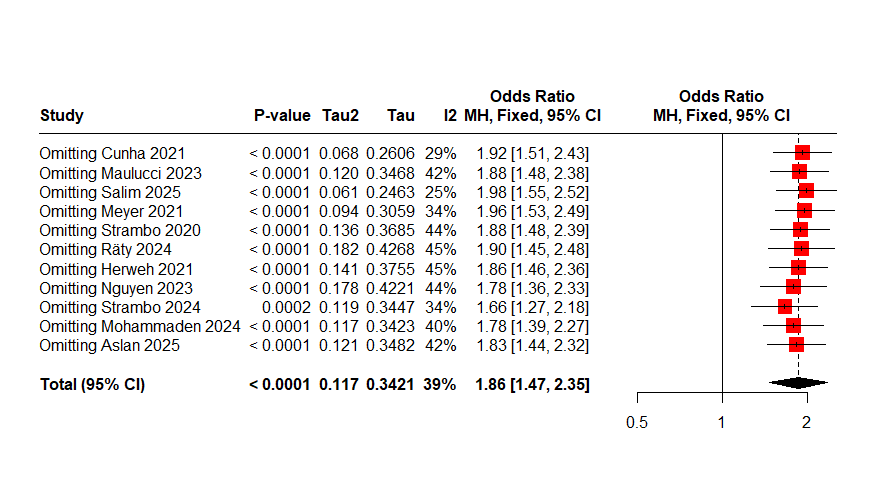


Supplementary Figure 11: Funnel plot for mortality rates at 90 days


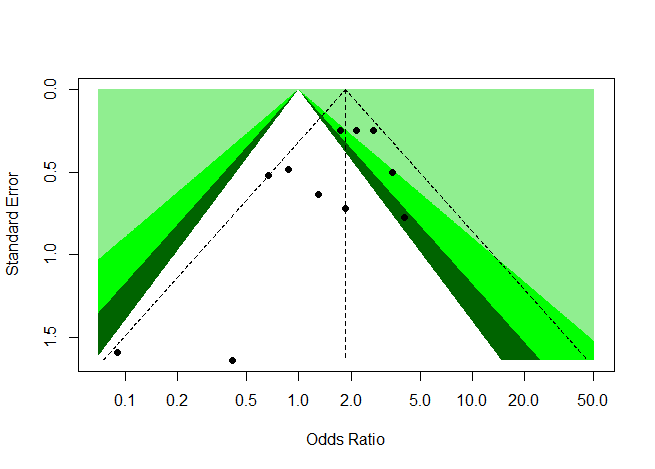


Supplementary Figure 12: Sensitivity analysis for change in NIHSS at 24 hours


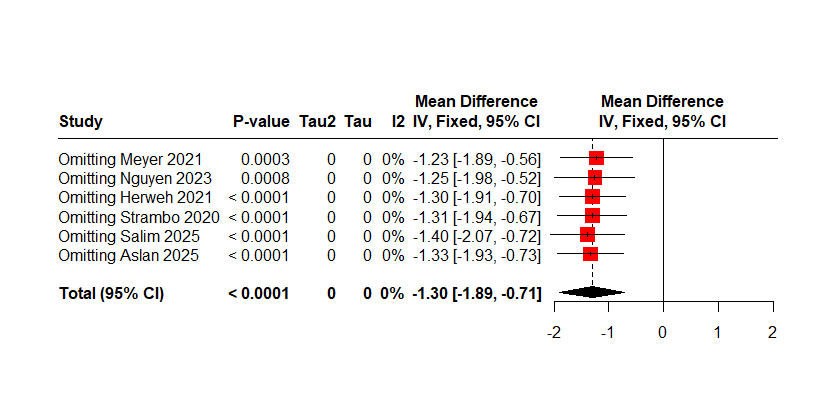


Supplementary Figure 13: Funnel plot for change in NIHSS at 24 hours


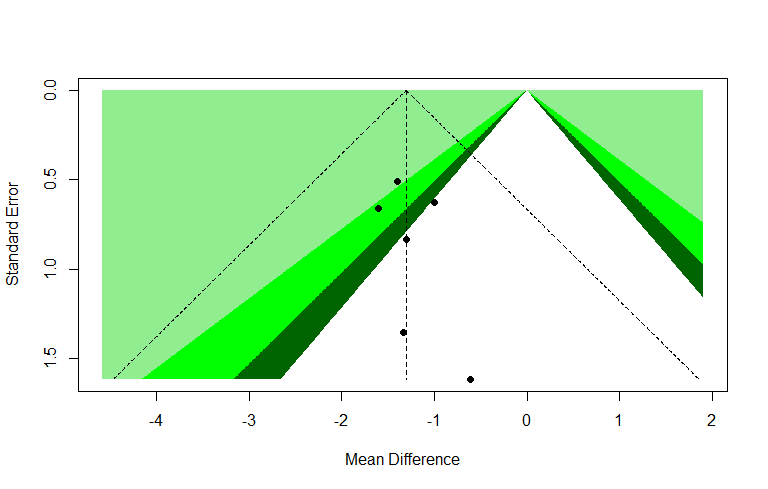


**Supplemetary Table 3: List of abbreviations**

| **Abbreviation** | **Full Form** |
| --- | --- |
| **BMM** | Best medical management |
| **BMT** | Best medical treatment *(standardized as BMM throughout manuscript)* |
| **CI** | Confidence interval |
| **ENI** | Early neurological improvement |
| **EVT** | Endovascular thrombectomy |
| **ICH** | Intracranial hemorrhage |
| **IVT** | Intravenous thrombolysis |
| **mRS** | Modified Rankin Scale |
| **NIHSS** | National Institutes of Health Stroke Scale |
| **OR** | Odds ratio |
| **PCA** | Posterior cerebral artery |
| **RCT** | Randomized controlled trial |
| **REML** | Restricted maximum likelihood |
| **sICH** | Symptomatic intracranial hemorrhage |
| **τ²** | Between-study variance (tau-squared) |
